# Maternal aging affects oocyte resilience to carbonyl cyanide-m-chlorophenylhydrazone -induced mitochondrial dysfunction in cows

**DOI:** 10.1371/journal.pone.0188099

**Published:** 2017-11-28

**Authors:** Kazuki Kansaku, Shun Takeo, Nobuhiko Itami, Airi Kin, Koumei Shirasuna, Takehito Kuwayama, Hisataka Iwata

**Affiliations:** Department of Animal Science, Tokyo University of Agriculture, Atsugi City, Kanagawa, Japan; University of Florida, UNITED STATES

## Abstract

Mitochondrial quality control is important for maintaining cellular and oocyte viability. In addition, aging affects mitochondrial quality in many cell types. In the present study, we examined how aging affects oocyte mitochondrial biogenesis and degeneration in response to induced mitochondrial dysfunction. Cumulus oocyte complexes were harvested from the ovaries of young (21‒45 months) and aged (≥120 months) cows and treated for 2 hours with 10 μM carbonyl cyanide-*m*- chlorophenylhydrazone (CCCP), or a vehicle control, after which cumulus oocyte complexes were subjected to *in vitro* fertilization and culture. CCCP treatment reduced ATP content and increased reactive oxygen species (ROS) levels in the oocytes of both young and aged cows. When CCCP-treated cumulus oocyte complexes were subsequently cultured for 19 hours and/or subjected to fertilization, high ROS levels in oocytes and a low rate of blastocyst development was observed in oocytes derived from aged cows. In addition, we observed differential responses in mitochondrial biogenesis to CCCP treatment between young and aged cows. CCCP treatment enhanced mitochondrial biogenesis concomitant with upregulation of SIRT1 expression in oocytes of young, but not aged, cows. In conclusion, aging affects mitochondrial quality control and recuperation of oocytes following CCCP-induced mitochondrial dysfunction.

## Introduction

Mitochondria are master regulators of energy generation and play versatile roles in apoptosis, calcium storage, and hormone synthesis [1]. Mitochondrial number and function are vital for oocyte development because oocytes containing high ATP content are responsible for increased developmental ability in cows [2] and interference with mitochondrial energy generation or function by carbonyl cyanide *p*-(trifluoromethoxy) phenylhydrazone treatment or photosensitization results in abnormal nuclear maturation and compromised developmental competence, respectively [3–5]. Oocyte quality declines with maternal age [6–9] and age-associated mitochondrial deterioration is closely related to a decline in oocyte quality [10,11]. In addition, decreases in mitochondrial DNA (MtDNA) copy number and ATP content and increases in reactive oxygen species (ROS) levels in oocytes have been reported in aged horses, hamsters, and mice [12–14]. In accordance with these findings, we previously reported that oocytes derived from aged cows exhibit high ROS levels, with decreased MtDNA copy number and developmental ability [15–17]. Mitochondrial quality in somatic cells is controlled by well-orchestrated processes of *de novo* synthesis, degeneration, fusion, and fission [18,19]. In somatic cells, upon treatment with the mitochondrial uncoupler carbonyl cyanide-*m*-chlorophenylhydrazone (CCCP), mitochondrial degeneration through the ubiquitin-proteasome system is enhanced [20–22]. In porcine oocytes, treatment with CCCP reduces ATP content, increases the expression of sirtuin 1 (SIRT1) and phosphorylated AMP activated protein kinase (pAMPK), and induces mitochondrial generation and degeneration in oocytes, as determined by the kinetics of the MtDNA copy number dynamics, the activation of *TFAM* expression, and mitophagy markers [23]. On the basis these findings, we suggest that dysfunctional mitochondria may enhance SIRT1 and AMPK activity as well as mitochondrial replenishment through *de novo* synthesis and degeneration in oocytes. This suggestion is supported by a study demonstrating that treatment of porcine oocytes with resveratrol enhances SIRT1 expression and induces both mitochondrial biogenesis and degradation, resulting in improved oocyte development [24]. Furthermore, the beneficial effect of resveratrol on SIRT1 activation, mitochondrial function, and developmental ability was found in oocytes harvested from young cows [25]. From these results, it has been hypothesized that induction of mitochondrial biogenesis and degeneration is a potentially useful countermeasure against age-associated oocyte deterioration. However, age-associated declines in the mitochondrial quality-control systems have been reported in human fibroblasts and murine pulmonary fibroblasts [26,27]. Therefore, in the present study, we treated oocytes derived from young and aged cows with CCCP, and subsequently subjected them to *in vitro* maturation. We then assayed the ATP content, SIRT1 expression, ROS levels, mitochondrial biogenesis, and blastocyst developmental ability between the two age groups. Our study provides additional insights into the role of age-associated deterioration of mitochondrial quality-control systems is present in bovine oocytes.

## Materials and methods

### Reagents and media

All chemicals were purchased from Nacalai Tesque (Kyoto, Japan), unless otherwise stated. CCCP and MG132 were obtained from Sigma Aldrich (St. Louis, MO, USA) and diluted in dimethyl sulfoxide (DMSO) at a concentration of 10 mM (1000×). *In vitro* maturation (IVM) was performed in medium 199 (Invitrogen, Carlsbad, CA, USA) supplemented with 10% fetal calf serum (FCS; 5703H; ICN Pharmaceuticals, Costa Mesa, CA, USA) and 5 mM taurine. The media used for *in vitro* fertilization (IVF) and *in vitro* culture (IVC) were based on modified-synthetic oviduct fluid (m-SOF) medium [28]. IVF medium was comprised of m-SOF medium supplemented with 0.4% fatty acid-free bovine serum albumin (BSA) and 10 IU/mL heparin. For IVC the medium was comprised of m-SOF supplemented with amino acids (Sigma Aldrich) and 1.5 mM glucose. The serum concentration of IVC medium was 1% for 0‒48 h post-IVF and 5% from 48 to 144 h post-IVF. IVM, IVF, and IVC for 0‒48h post IVF was conducted at 38.5°C under 5% CO_2_ and 95% air, and IVC for 48-144h post-IVF was conducted under 5% CO_2_, 5% O_2_ and 90% N_2_.

### Definition of young and aged cows

We previously reported age-associated deterioration of oocyte quality as determined by nuclear maturation, fertilization rate, mitochondrial DNA copy number, and rate of development to the blastocyst stage as well as comprehensive gene expression analysis when the age of donor cows exceeded 120 months [15,17,29]. In this study, we defined oocytes harvested from cows that were more than 120 months old as the aged oocyte group and those collected from cows that were 21‒45 months old as the young oocyte group.

### Ovary and oocyte collection

Bovine ovaries with a functional corpus luteum and large dominant follicles (>10 mm in diameter) were harvested from Japanese black cows (*Bos taurus*) at an abattoir, and were transported to the laboratory in phosphate-buffered saline (PBS) at approximately 25°C. The ovaries were discarded, and thus this study was approved by the Ethical Committee for Animal Experiment of Tokyo University of Agriculture. Cumulus oocyte complexes (COCs) were aspirated from antral follicles (3‒6 mm in diameter) using a 21-gauge needle connected to a 5 mL syringe (Terumo, Tokyo, Japan). COCs with thick, compact cumulus cells and oocytes with homogeneous cytoplasm were used for experiments.

### CCCP treatment and *in vitro* maturation

COCs were cultured in IVM medium containing either the vehicle (DMSO) or 10 μM CCCP for 2 h. After treatment, COCs were washed and cultured in 100 μL IVM medium (10 oocytes/drop) under paraffin oil for 19 h. The duration of CCCP treatment was determined according to a previous report [23].

### *In vitro* fertilization and embryo culture

*In vitro* fertilization of oocytes was conducted as previously described [16]. Briefly, thawed semen from a Japanese Black bull was washed with 30‒60% Percoll discontinuous gradient solution (Amersham Co., Ltd., Uppsala, Sweden) by centrifugation for 10 min (800 × *g*). Oocytes and semen were incubated for 6 h (10 oocytes/100 μL drop), then presumptive zygotes were subsequently cultured for seven days and the rate of development to the blastocyst stage and the total cell number of blastocyst-stage embryos were examined. To assay the cell number blastocysts were fixed with 4% paraformaldehyde (Funakoshi, Tokyo, Japan) in PBS for 24 h, and then mounted with antifade solution containing 4′,6-diamidino-2-phenylindole (Pro-long gold antifade reagent with DAPI; Invitrogen) on glass slides following permeabilization with PBS-polyvinyl alcohol (PVA) containing 0.25% Triton X-100 for 30 min. The blastocysts were observed under a digital fluorescence microscope (Olympus, Tokyo, Japan).

### ATP measurements

ATP content of individual oocytes was measured as luminescence generated in an ATP-dependent luciferin–luciferase bioluminescence assay (ATP assay kit; Toyo-Inc., Tokyo, Japan), as previously described [16]. Luminescence was measured with a luminometer (Gene Light 55; Microtech, Chiba, Japan).

### Immunostaining

Immunostaining of oocytes was conducted as previously described [25,30]. Briefly, oocytes were fixed in 4% paraformaldehyde-PBS for 12 h, then permeabilized with PBS-PVA containing 0.25% Triton X-100 for 30 min at room temperature, followed by 1 h of incubation in blocking solution (PBS containing 5% BSA, 1% Tween 20, and 5% goat serum; GIBCO BRL, Paisley, UK). The primary antibodies used were rabbit polyclonal antibodies against SIRT1 (1:200, Santa Cruz Biotechnology, Santa Cruz, CA) and Ubiquitin (1:200, Cell signaling Technology, Inc. San Diego, CA, USA) and the secondary antibody used was anti-rabbit IgG (H + L), F (ab’)_2_ fragment (Alexa Fluor555 Conjugate; 1:1,000; Cell Signaling Technology, Inc. San Diego, CA, USA). The oocytes were mounted on glass slides with antifade solution containing DAPI and observed under a digital fluorescence microscope (BZ-8000; Keyence, Tokyo, Japan). Fluorescence intensity was analyzed with Image J software (NIH, Bethesda, MD, USA). A negative control was obtained using the procedure described above without addition of the primary antibody. Effectiveness of the immunostaining of SIRT1 was confirmed using a neutralization test in in which oocytes were cultured with the primary antibody (2 μg/mL IgG) or the primary antibody and a SIRT1-peptide (Abcam 7770–100, 2 μg/mL or 10 μg/mL) and fluorescent intensity significantly was found to be significantly decreased in a peptide-concentration-dependent manner (S1 Fig).

### Measurement of ROS levels

Oocyte ROS levels were measured using CellROX Green Reagent (Invitrogen) according to the manufacturer’s protocol. Briefly, COCs were denuded of cumulus cells by vortexing and oocytes were treated CellROX Reagent for 30 min. Fluorescence images were captured under a fluorescent digital microscope (BZ-8000; Keyence) and fluorescence intensity was measured with Image J software (NIH).

### Assessment of mitochondrial DNA copy number

Individual denuded oocytes were lysed in 6 μL of lysis buffer (20 mM Tris, 0.4 mg/mL proteinase K, 0.9% Nonidet-40, and 0.9% Tween 20) at 55°C for 30 min, followed by incubation at 98°C for 5 min. MtDNA copy number was then determined by real-time PCR using a Rotor-Gene 6500 real-time rotary analyzer (Corbett Research, Sydney, Australia) as described previously [15]. Primers (5′-ATTTACAGCAATATGCGCCC-3′ and 5′-AAAAGGCGTGGGTACAGATG-3′) were designed using Primer-BLAST for the bovine mitochondrial gene ND5 (LOCUS NC006853; 1.82-kb region from base 12109 to base 13929). PCR was performed with initial denaturation at 95°C for 3 min, followed by 40 cycles at 98°C for 5 s, and 59°C for 11 s. SYBR green fluorescence was measured at the end of each extension step. A standard curve was generated for each assay using 10-fold serial dilutions representing copies of the external standard, which was the PCR product of the corresponding gene cloned into a vector using the Zero Blunt TOPO PCR cloning kit (Invitrogen). The product was sequenced for confirmation prior to use.

### Logic of evaluation of mitochondrial biogenesis and degradation

**Fig 1:**
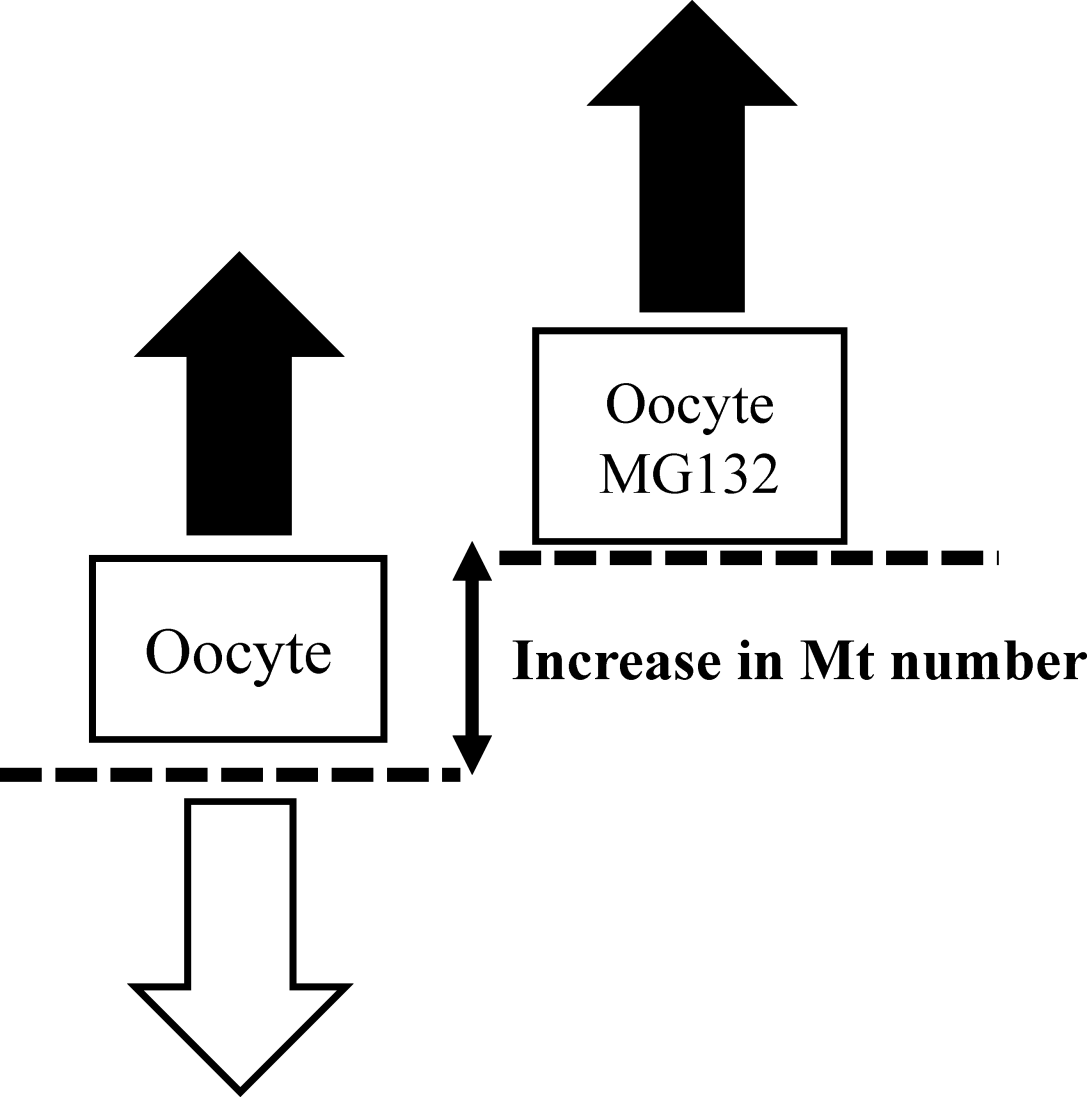
Evaluation of mitochondrial biogenesis and degradation by using MG132. COCs were cultured in medium containing vehicle or MG132 for 21 h. In control medium (left), mitochondrial DNA copy number in oocytes reflect both mitochondrial biogenesis (black arrow) and degradation (white arrow) in oocytes. In the medium containing MG132 (right), mitochondrial DNA copy number in oocytes reflects mitochondrial biogenesis (black arrow) because mitochondrial degradation is inhibited by MG132. Comparison between the two conditions allows for prediction of how mitochondrial biogenesis generation occurs in oocytes.

**Fig 2:**
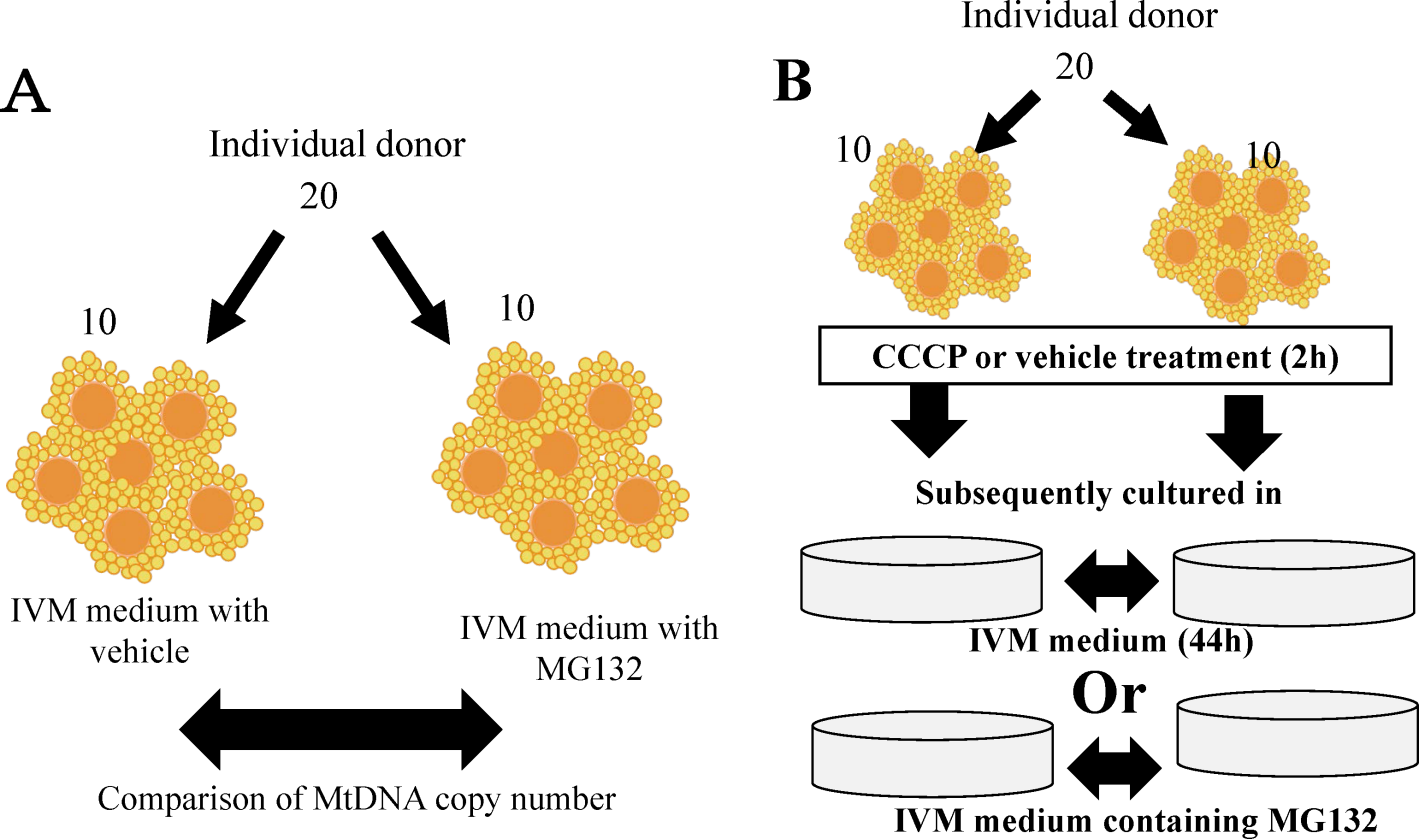
Effect of MG132 and CCCP on MtDNA copy number in oocytes. (A) Twenty COCs collected from individual cows were divided into two groups, and incubated in medium containing vehicle or 10 μM MG132 for 21 h, after which mitochondrial DNA copy number in oocytes was assayed. (B) Twenty COCs collected from individual cows were divided into two groups and treated with the vehicle or 10 μM CCCP for 2 h, then cultured for 19 h in IVM medium. Two IVM media were used in this experiment; an IVM medium where both mitochondrial biogenesis and degradation occur and an IVM medium containing MG132 in which only mitochondrial biogenesis occurs.

Mitochondrial generation and degeneration both occur in oocytes. Therefore, the MtDNA copy number at the end of the culture period does not reflect intrinsic mitochondrial generation. Yoshi et al. showed that after CCCP treatment, the mitochondrial outer membrane was digested by the proteasome followed by degeneration through mitophagy [31]. In addition, it has been shown that MG132, a proteasome inhibitor, inhibited mitochondrial degeneration in porcine oocytes [24,23]. In the present study, we used medium containing MG132 or vehicle which allows only mitochondrial biogenesis (Fig 1 right; presence of only mitochondrial biogenesis results in an increase in mitochondrial content) or allows both mitochondrial biogenesis and degradation (Fig 1 left; presence of both biogenesis and degradation results in constant mitochondrial content). Comparison between the two conditions allows for prediction of how mitochondrial biogenesis and degradation occur in oocytes. Furthermore, it is known that there are extensive differences in MtDNA copy number in oocytes among donor cows, but the mitochondrial DNA copy number predicted by 10 cohorts oocytes was consistent for each donor gilt and cow [15,24]. Then, MtDNA copy numbers were compared between samples obtained from each donor cow. Twenty COCs were collected from each donor cow and divided into two groups of cohort oocytes: one group of oocytes were treated with drug and others were treated with vehicle (Fig 2).

## Experimental design

### Effect of CCCP on ATP content

COCs were randomly selected from pooled COCs derived from young and aged cows, and were equally divided into two groups, which were incubated in medium containing either the vehicle or 10 μM CCCP for 2 h. After the CCCP treatment, oocytes were denuded from cumulus cells, and the oocyte ATP content was examined. Thenumber of cows and oocytes used in this experiment is presented in Table 1.

**Table 1:** Number of donor and oocytes used in experiments.

Experiment	Aged	No. of	Month ±	No. of
		cows	SEM	oocytes
ATP content	Young	8	28.9±0.5	142
	Aged	8	160.8±6.2	141
Development	Young	10	26.6 ± 0.5	200
	Aged	10	150.7 ± 7.0	200
ROS content	Young	10	26.7 ± 0.7	98
	Aged	10	158.2 ± 7.7	108
Immunostaining	Young	7	26.9 ± 0.7	38
	Aged	7	165.9 ± 7.0	45
Mt-number	Young	13	26.9 ± 0.5	260
Effect of MG132	Aged	13	144.6 ± 4.7	260
Mt-number	Young	10	27.5 ± 1.3	200
Control medium	Aged	10	153.8 ± 6.8	200
Mt-number	Young	12	34.1 ± 2.9	240
MG132 medium	Aged	13	158.1 ± 5.5	260
SIRT1 expression	Young	32	26.4 ± 0.4	197
	Aged	25	154.3 ± 4.6	202

### Effect of CCCP on oocyte development

We compared the effect of CCCP treatment on the developmental ability of oocytes between young and aged cows. The developmental rate of oocytes differed among donors, thus the effect of CCCP treatment was evaluated within oocytes derived from individual cows. Twenty COCs were collected from individual young and aged cows and were divided into two groups, which were treated with either the vehicle or 10 μM CCCP for 2 h and then subjected to IVM, IVF, and IVC. The rate of development to the blastocyst stage and the total cell number of the blastocysts were examined within the cohort of oocytes. Oocytes from 10 young and aged donor cows were used (Table 1).

### Effect of CCCP on ROS content in oocytes

Here, we compared the ROS content in CCCP-treated and untreated oocytes. COCs were randomly selected from pooled COCs harvested from young or aged cows, and treated with CCCP (vehicle or 10 μM) for 2 h and then cultured in IVM medium for 19 h. Just after CCCP treatment or following restoration culture (19 h), the oocyte ROS content was determined. Ten young and aged cows were used as donors and randomly used in this experiment (Table 1).

### Effect of inhibition of protein degradation on mitochondrial content in oocytes

We examined whether MG132 inhibited protein degradation and increased mitochondrial content in oocytes. Randomly selected COCs derived from 7 young and 7 aged cows (Table 1) were incubated with the vehicle or 10 μM MG132 for 21 h, and levels of ubiquitinated protein were examined by immunostaining, as described above. In the next experiment, we examined the effect of MG132 on oocyte MtDNA copy number. As shown in Fig 2A, 20 oocytes were collected from individual young and aged cows (Table 1), the cohort oocytes were incubated in medium with or without 10 μM MG132 for 21 h, and then MtDNA copy number was compared between MG132-treated and vehicle-treated oocytes within the same donor.

### Effect of CCCP treatment on mitochondrial content

We compared the CCCP treatment-induced mitochondrial generation and degeneration in oocytes between young and aged cows. Twenty COCs collected from individual young or aged cows were divided into two groups, and the COCs were treated with the vehicle or 10 μM CCCP for 2 h, after which the oocytes were cultured in IVM medium for 19 h, followed by MtDNA copy number measurement (Fig 2B). As described in evaluation of mitochondrial biogenesis anddegradation section, this experiment used two types of IVM media (IVM or IVM medium containing MG132). COCs of 10 young and 10 aged cows were used for IVM medium following CCCP treatment, and COCs of 12 young and 13 aged cows were used for IVM medium containing MG132 (Table 1).

### Effect of CCCP treatment on SIRT1 expression in oocytes

We compared the CCCP treatment-induced changes in SIRT1 expression levels in oocytes between young and aged cows. Approximately 20 COCs were randomly selected from pooled COCs harvested from young and aged cows (Table 1), and divided into two groups that were treated with either the vehicle or 10 μM of CCCP for 2 h, and then, the SIRT1 levels were examined immediately or 3 h after treatment. This experiment was repeated three times.

### Statistical analysis

Comparisons of data among three or more groups were performed using an analysis of variance (ANOVA) test followed by Tukey’s post-hoc tests. Comparisons between two groups were conducted by Student’s *t* test. Percentages were arcsine-transformed prior to analyses. Developmental rates were compared by the Chi-square tests. Values of P < 0.05 were considered to indicate statistical significance.

## Results

### CCCP treatment decreased oocyte ATP content

**Fig 3:**
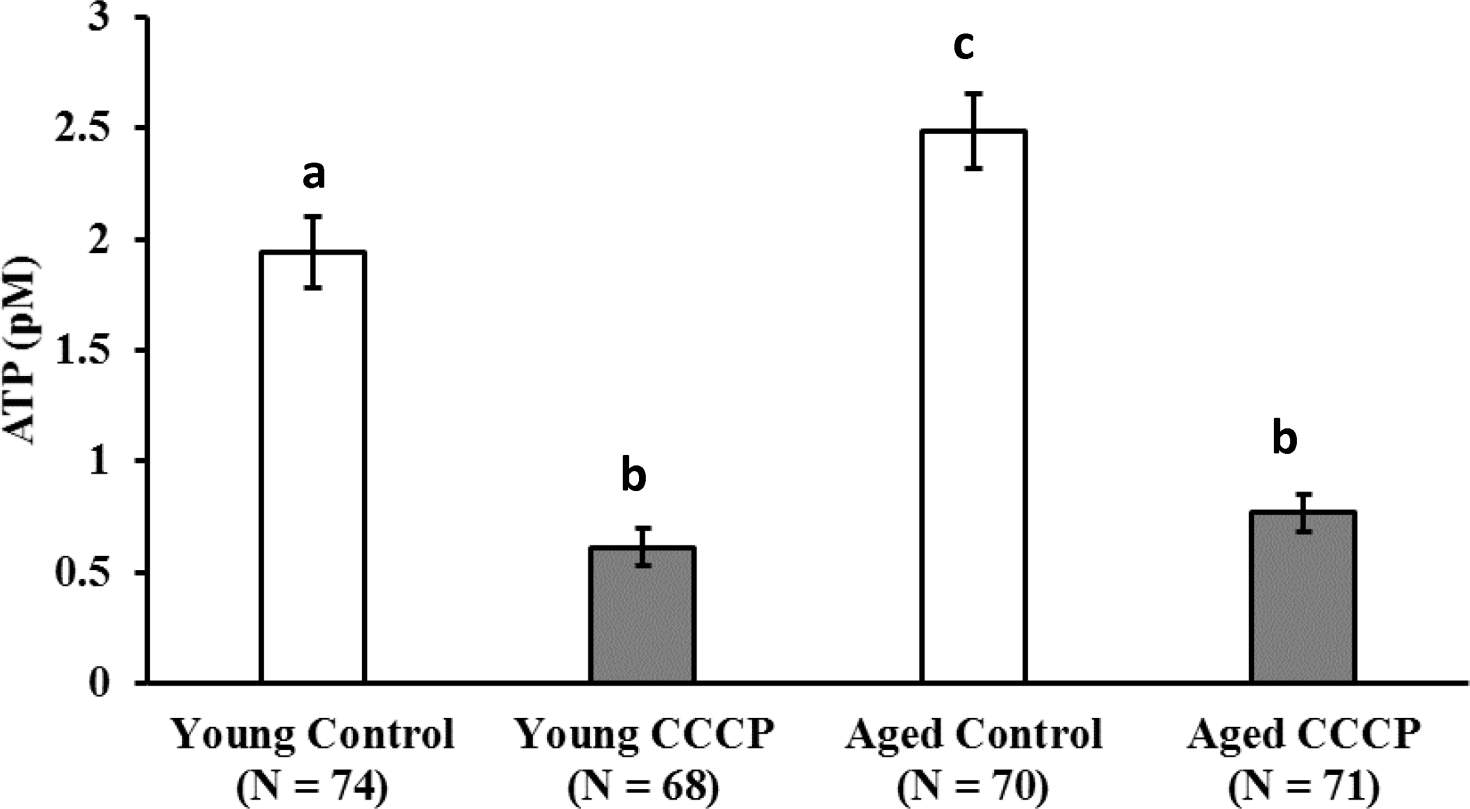
Effect of CCCP treatment on ATP content of oocytes derived from young and aged cows. COCs from young and aged cows were treated with CCCP or vehicle (Control) for 2 h, and then ATP content in the oocyte was determined. Data are presented as mean ± SE. a−c indicate statisticaly significantly differences, P < 0.001. (N = 8, described in Table 1).

The ATP content of oocytes treated with or without CCCP is shown in Fig 3. CCCP is a potent mitochondrial membrane uncoupler. CCCP treatment significantly decreased oocyte ATP content in both the young and aged groups (Fig 3: young; vehicle vs. CCCP: 1.9 ± 0.16 vs. 0.61 ± 0.08, aged; vehicle vs. CCCP: 2.5 ± 0.17 vs. 0.77 ± .0.08, P < 0.001).

### CCCP treatment decreased oocyte developmental competence in aged cows

We examined how CCCP treatment affect developmental ability of oocytes derived from young and aged cows. CCCP treatment did not affect the developmental rate of oocytes derived from young cows (blasturation rate % control vs. CCCP: 46.0 ± 5.6 vs. 44.0 ± 4.0, Table 2), but it significantly reduced the developmental ability of oocytes derived from aged cows (control vs. CCCP: 35.0 ± 6.1 vs. 17.0 ± 4.2, Table 2, P < 0.01). Total blastocyst cell numbers were not significantly different between the two treatment groups. Oocytes were treated with CCCP or vehicle and after fertilization, oocytes were cultured for 7 days at which rate of development to the blastocyst stage and total cell number (TCN) of the blastocyst were determined. Table 2. Effect of CCCP treatment on developmental competence in oocytes derived from young and aged cows.

**Table 2:** Effect of CCCP treatment on developmental competence in oocytes derived from young and aged cows.

Age groups	CCCP	No. of trials	No. of cows	No. of oocytes	Mean ± SEM					
	10μM				Blastocyst(%)			TCN		
Young	-	10	10	100	46.0	±	5.6	77.8	±	4.3
	+	10	10	100	44.0	±	4.0	76.7	±	3.9
Aged	-	10	10	100	35.0	±	6.1b	88.6	±	8.5
	+	10	10	100	17.0	±	4.2a	82.8	±	13.0

a-b; (P<0.05)

### ROS content in oocytes was high in aged cows 19 hours after CCCP treatment

**Fig 4:**
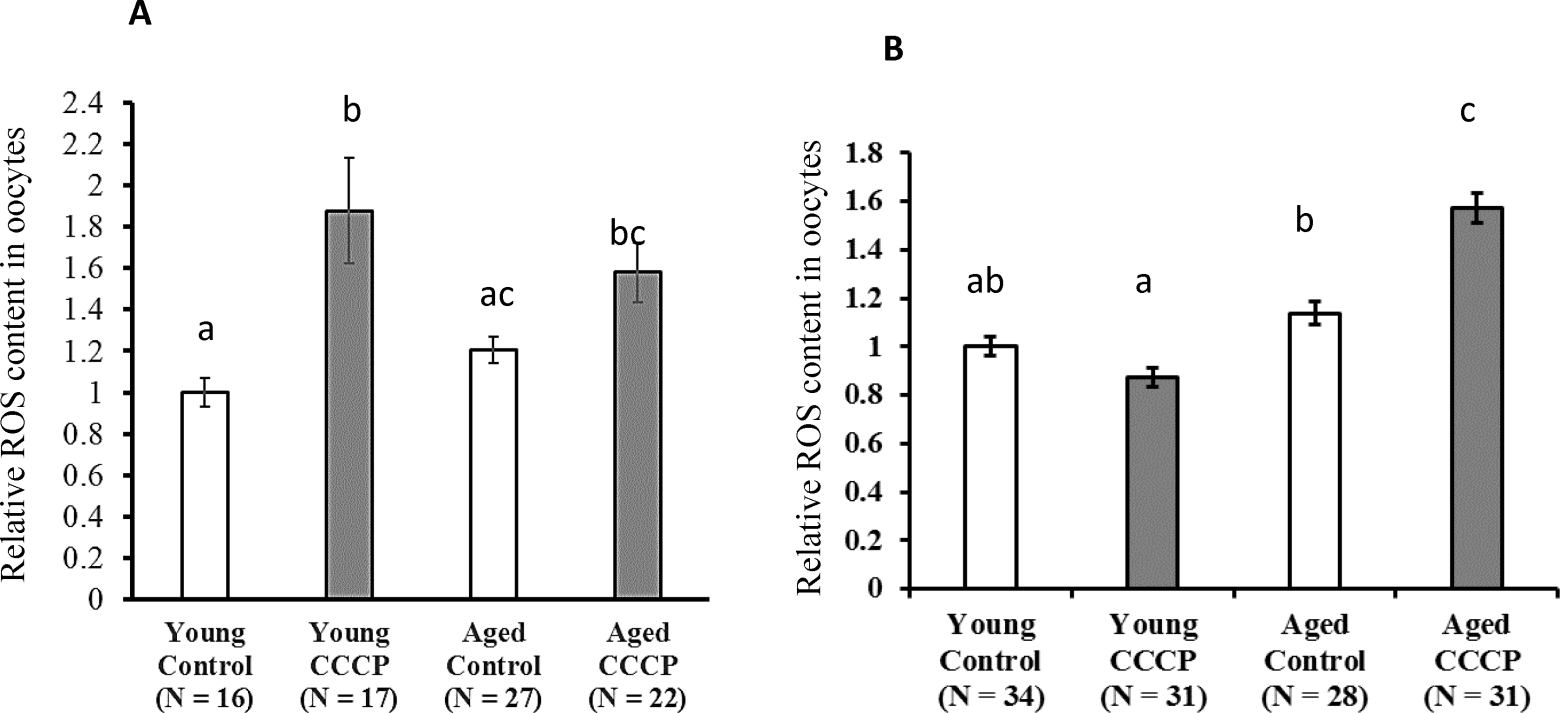
Effect of CCCP treatment on ROS levels in oocytes derived from young and aged cows. Oocytes were treated with CCCP or vehicle (Control) and cultured for 19 h. ROS content was assayed immediately after CCCP treatment or following restoration culture (19 h). (A) ROS content in oocytes. Y axis: relative ROS content in oocytes. Average fluorescence intensity of vehicle-treated oocytes from young cows was set to 1.0 and values represent the fold difference in fluorescence intensity. Data is expressed as mean ± SE. a−c, P < 0.05. (B) Representative fluorescent images of cultured oocytes treated with or without CCCP treatment followed by staining with ROS detection reagents are shown. (N = 7, described in Table 1).

CCCP differentially affect oocytes developmental ability and we evaluated mitochondrial quality of oocytes following CCCP treatment between young and aged cows. As shown in Fig 4A, CCCP induced high ROS levels in the young group immediately after CCCP treatment, but the difference did not reach statistical significance in the aged group. However, pair-wise comparison between CCCP-treated oocytes and vehicle treated oocytes revealed CCCP significantly induced ROS generation in oocytes of aged cows (Student’s *t*-test, *P* = 0.011). After 19 h of restoration culture, in oocytes of young cows, ROS content in oocytes was almost similar between CCCP treated and untreated groups, whereas in aged cows CCCP-treated oocytes had significantly higher levels of ROS compared to those in untreated oocytes (Fig 4B).

### MG132 treatment inhibited mitochondrial degeneration in oocytes of young, but not aged, cows

**Fig 5:**
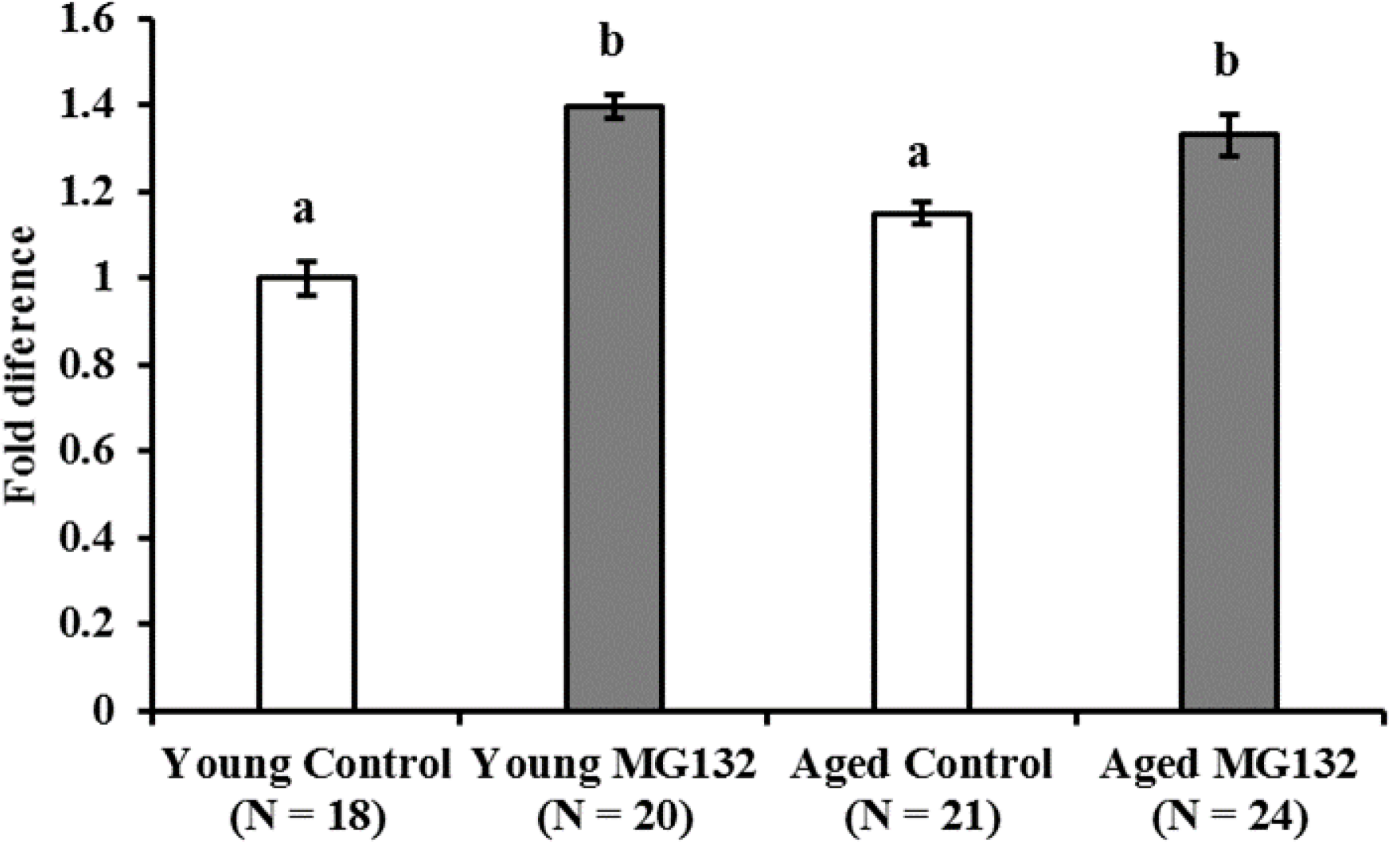
Effect of MG132 on ubiquitinated protein levels in oocytes. Oocytes from young and aged cows were incubated with or without MG132 for 21 h and then immunostained for ubiquitinated proteins. Average fluorescence intensity of oocytes is shown with values of vehicle-treated oocytes from young cows being set to 1.0 (Y axis). Data are expressed as mean ± SE. a-b, P < 0.05. (N = 7, described in Table 1).

**Fig 6:**
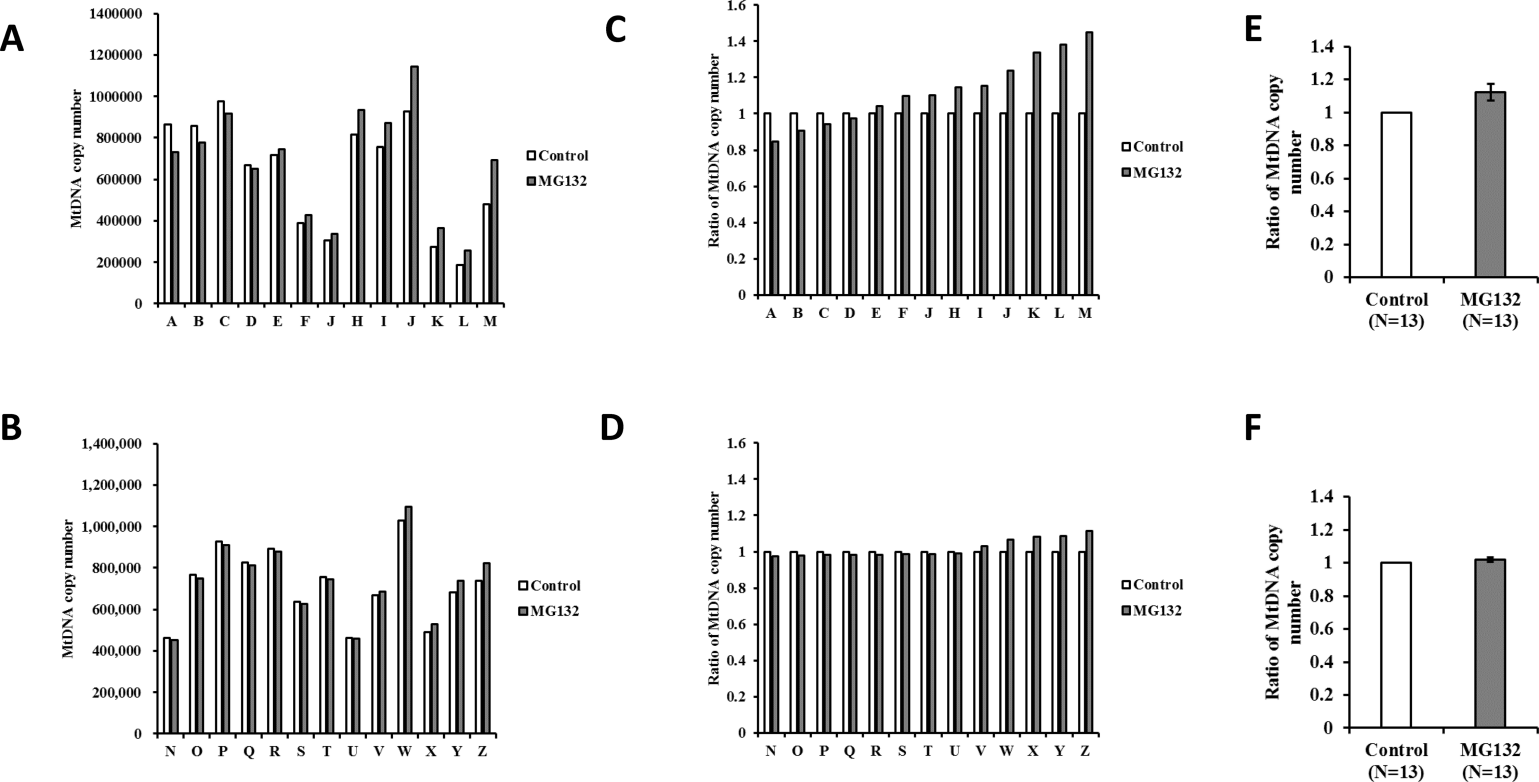
Effect of MG132 treatment on MtDNA copy number in oocytes derived from young and aged cows. Oocytes were collected from individual cows, and were cultured with (dark bars) or without MG132 (white bars) for 21 h, and then oocyte MtDNA copy number was examined. (A and B) Average mitochondrial DNA copy number in oocyte for each donor cows (young cows; A−M, aged cows; N−Z). (C and D) Relative MtDNA copy number in which mitochondrial DNA copy number in vehicle treated oocytes of each donor were set to 1.0. (E-F) Average of relative mitochondrial DNA copy number in MG132 treated oocytes to that of vehicle treated oocytes. Data are expressed as mean ± SE. a-b, P < 0.05. (N = 13. described in Table 1).

Proteasome inhibitor inhibited protein degradation in oocytes and increased mitochondrial copy number resulting from the loss of mitochondrial degradation. As shown in Fig 5, MG132 significantly increased the level of ubiquitinated protein in oocytes of both age groups (1.4 and 1.14-fold for young and aged cows, respectively, P < 0.05). The MtDNA copy number varied greatly among donors (Fig 6A and 6B). Therefore, we compared the relative MtDNA copy numbers in CCCP-treated and untreated oocytes within each donor group (Fig 6C and 6D). As summarized in Fig 6E and 6F, supplementation of IVM medium with 10 μM MG132 significantly increased the MtDNA copy number in the oocytes from young cows (1.12-fold, P < 0.05), whereas no differences were observed in the oocytes derived from aged cows.

### CCCP treatment increased mitochondrial biogenesis in oocytes of young, but not aged, cows

**Fig 7:**
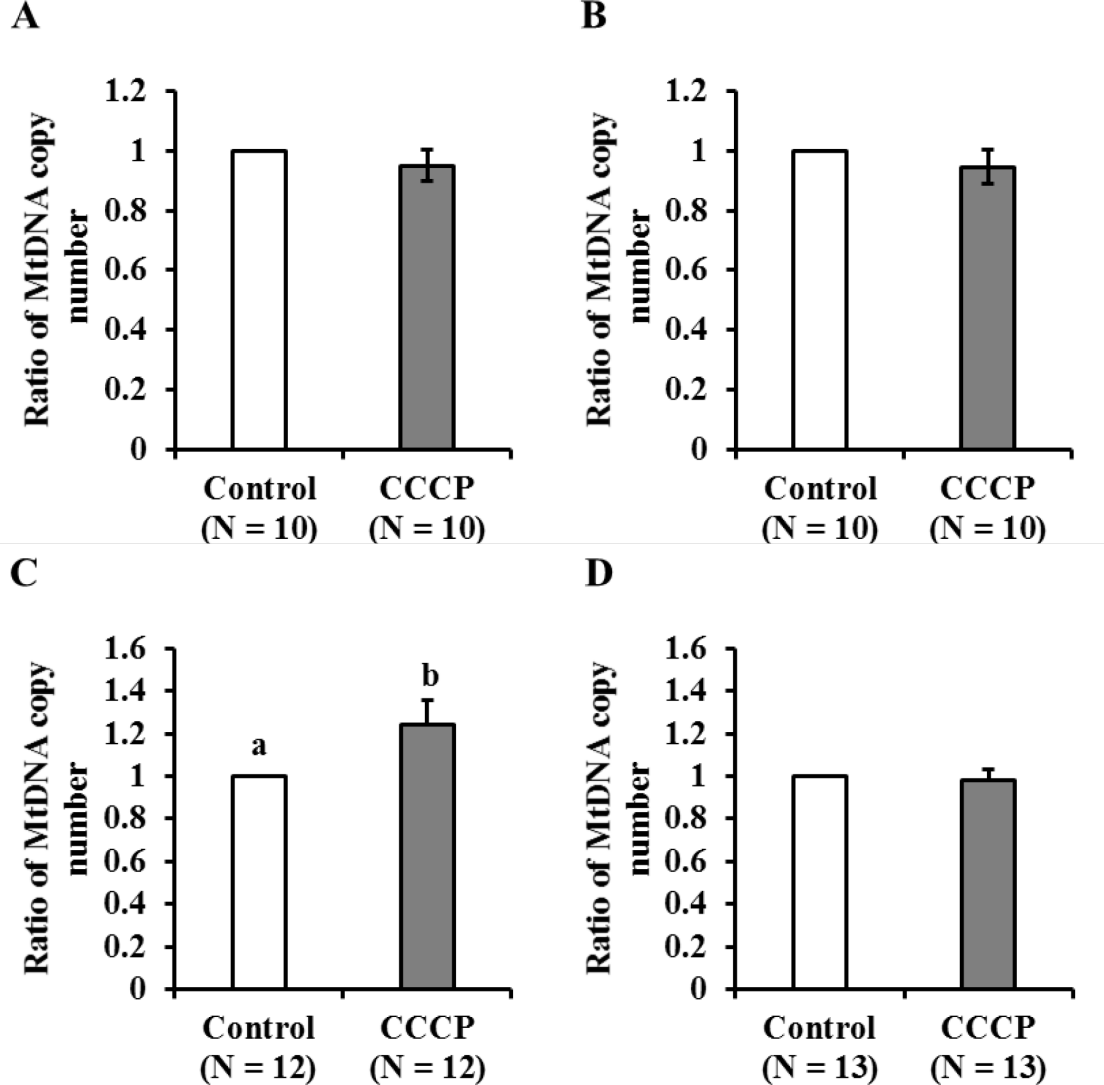
Ratio of MtDNA copy number of CCCP-treated to vehicle-treated oocytes derived from young and aged cows. Oocytes were collected from individual cows and were treated with CCCP (dark bars) or vehicle (white bars) and then cultured in IVM medium for 19 h. After incubation, the MtDNA copy number was assayed. A−B) After CCCP treatment, oocytes derived from young (A; N = 10) and aged cows (B; N = 10) were cultured in IVM medium. C−D) After CCCP treatment, oocytes derived from young (C; N = 12) and aged (D; N = 13) were cultured in medium containing MG132. Mitochondrial DNA copy number of vehicle-treated oocytes from each donor was set to 1.0 and the average of relative mitochondrial DNA copy number of CCCP-treated oocytes to that of vehicle-treated oocytes is presented. Data are expressed as mean ± SE. a-b, P < 0.05.

MG 132 inhibited mitochondrial degradation (Exp4), which allowed us to evaluate mitochondrial biogenesis, using medium containing MG132 or not. When CCCP-treated COCs were cultured in IVM medium containing only the vehicle, the MtDNA copy numbers did not differ between CCCP-treated and untreated oocytes, regardless of the age of the donor (Fig 7A). However, when CCCP-treated COCs were incubated in medium containing MG132, the MtDNA copy number significantly increased (1.24-fold) in oocytes derived from young, but not aged, cows (Fig 7B).

### Activation of SIRT1 in oocytes following CCCP treatment was observed in young, but not aged, cows

**Fig 8:**
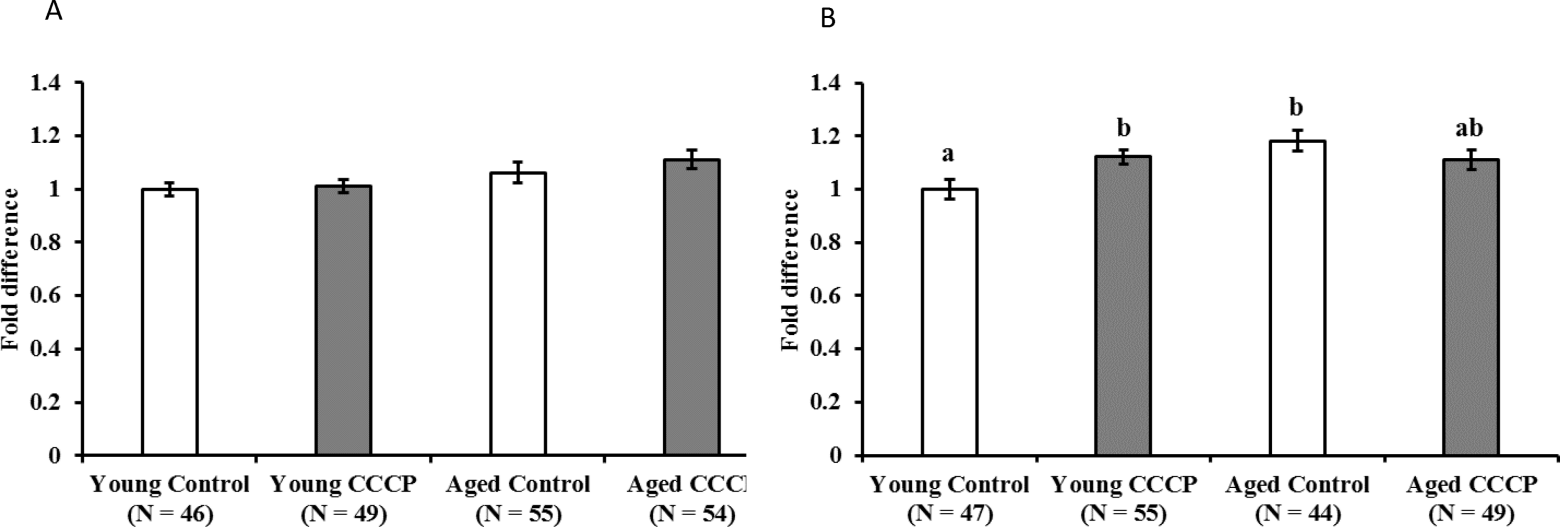
Effect of CCCP treatment on SIRT1 expression in oocytes. Oocytes were treated with CCCP for 2 h and then incubated in IVM medium. Immediately following CCCP treatment (A) and 3 h after treatment (B) oocytes were immunostained for SIRT1, and expression levels of fluorescent intensity were presented. Average expression levels of SIRT1 in vehicle treated oocytes from young cows were set to 1.0. Data are expressed as mean ± SE. a-b, P < 0.05. Oocyte from 32 young and 25 aged cows were used in this experiment.

SIRT1 is a potent upstream regulator of mitochondrial biogenesis regulating PGC1α and TFAM. [23, 32]. Low mitochondrial biogenesis in aged cows prompted us to examine whether SIRT1 expression levels following CCCP treatment differ between young and aged cows. Immediately following CCCP treatment, no differences were observed in SIRT1 levels between CCCP treated and untreated oocytes for either age group (Fig 8A). After 3 h of CCCP treatment, SIRT1 expression was higher in CCCP treated oocytes derived from young cows compared with those of their untreated oocytes (Fig 8B, control vs. CCCP: 1.00 ± 0.04 vs. 1.12 ± 0.03, P < 0.01), whereas no change was observed between CCCP treated and vehicle treated oocytes derived from aged cows. Furthermore, SIRT1 expression was higher in oocytes derived from aged cows than those from young cows.

## Discussion

In the present study, CCCP induced mitochondrial dysfunction, stimulated mitochondrial biosynthesis, and increased SIRT1 expression in oocytes obtained from young cows, and the developmental ability of oocytes was retained even after CCCP treatment. However, in oocytes obtained from aged cows, CCCP treatment did not upregulate mitochondrial biogenesis and expression of SIRT1, and their developmental ability was reduced following treatment. To the best of our knowledge, this is the first report to demonstrate an age-associated decline in resilience of mitochondria in oocytes.

**Fig 9:**
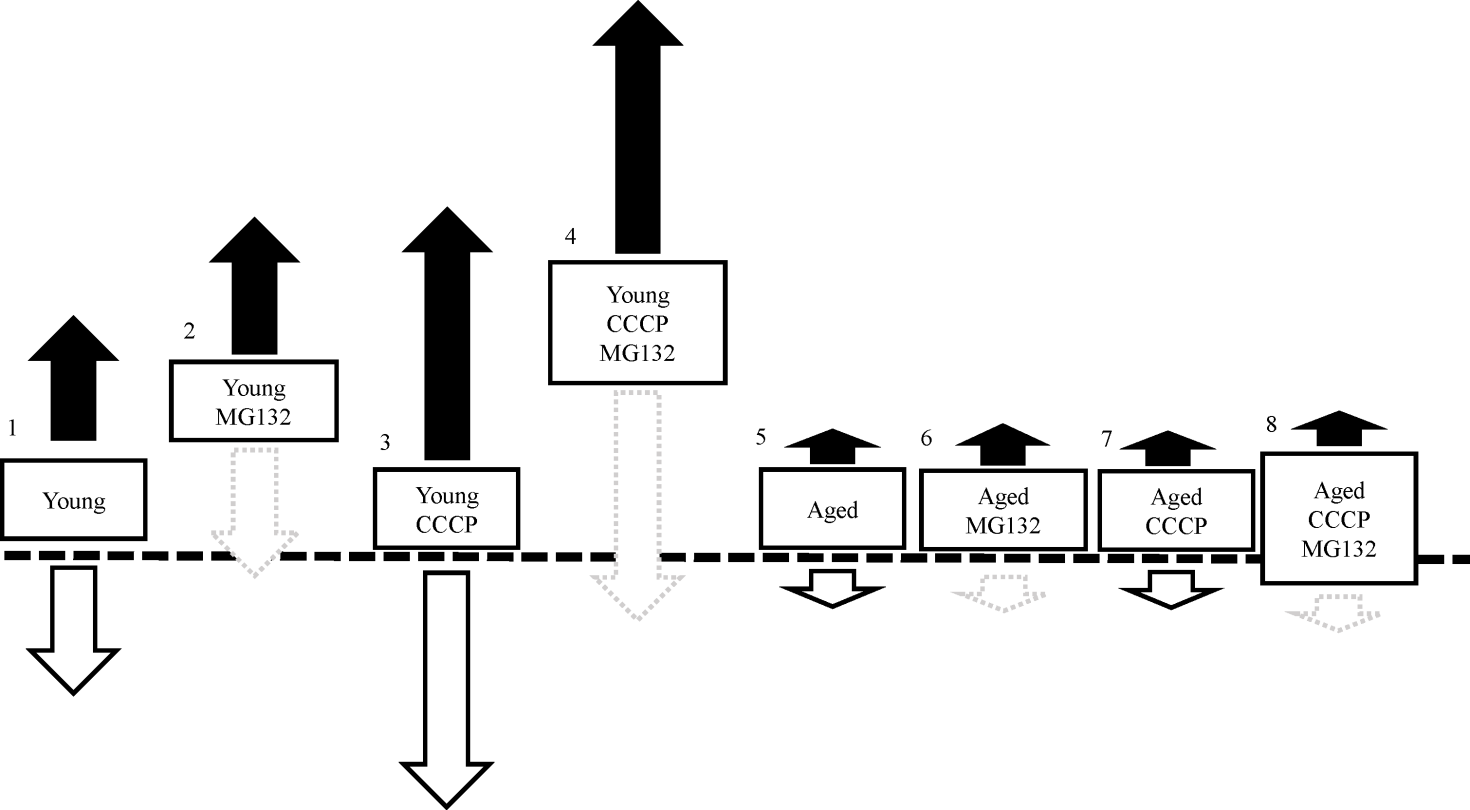
Summary of the effect of CCCP treatment on MtDNA copy number in oocytes. MG132, which inhibits mitochondrial degeneration, increased MtDNA copy number in the oocytes of young cows (1 vs. 2), but not in the oocytes of aged cows (5 vs. 6), indicating that there was less mitochondrial biogenesis and degeneration in the oocytes of aged cows. CCCP treatment of oocytes did not affect mitochondrial DNA copy number when oocytes were incubated in IVM medium without MG132 (1 vs. 3). However, in medium containing MG132, CCCP treatment upregulated mitochondrial biogenesis in the oocytes of young cows (2 vs. 4), but did not affect mitochondrial biogenesis in oocytes of aged cows (6 vs. 8). White arrows indicate degradation, black arrows indicate biogenesis and dotted arrows indicate inhibition of degeneration.

CCCP reduces cellular mitochondrial membrane potential and mitochondrial ATP generation [20–22, 33]. In the present study, 2-h CCCP treatment decreased oocyte ATP content compared with that in untreated (vehicle) oocytes. Consistent with this finding, our previous studies demonstrated that when the COCs of gilts were treated with CCCP, ATP content in oocytes decreased to one-third of the levels of that in untreated oocytes [23]. Furthermore, in subsequent experiments, treatment of denuded oocytes dramatically reduced ATP content in oocytes (young: pre-CCCP treatment 1.44 ± 0.2, after CCCP treatment 0.13 ± 0.02; aged: pre-CCCP treatment 1.34 ± 0.22, after CCCP treatment 0.30 ± 0.11, P < 0.01, S2 Fig). Supporting this data, we recently showed in pigs that CCCP reduced ATP content in oocytes, but not in granulosa cells, likely because oocytes largely relay on oxidative phosphorylation while granulosa utilize glycolysis [34]. These results indicate that CCCP directly affects oocyte mitochondria. In the present study, the developmental ability to reach the blastocyst stage was similar between CCCP-treated and untreated oocytes derived from young cows. Consistent with this result, CCCP-treated gilts oocytes were preciously founded to exhibit low ATP content immediately following treatment, but had developmental rates to reach the blastocyst stage similar to that of vehicle-treated oocytes following *in vitro* maturation and parthenogenetic activation [23]. However, we demonstrated that this is not the case in oocytes derived from aged cows. Our results suggest that the ability of oocytes and/or COCs to recuperate from mitochondrial dysfunction is decreased in oocytes derived from aged cows compared with that in oocytes from young cows. To test this hypothesis, we examined the ROS content in oocytes immediately after CCCP treatment or after 19 hours of incubation following CCCP treatment. We found that CCCP treatment induced ROS generation just after CCCP treatment in both young and aged groups. However, after 19h of culture, ROS content was higher in CCCP-treated oocytes derived from aged cows than in the vehicle-treated oocytes, whereas no difference was observed between CCCP-treated and untreated oocytes derived from young cows. This suggests that oocytes from aged cows have less ability to recover from CCCP induced mitochondrial dysfunction. Furthermore, ROS content in vehicle-treated oocytes derived from aged cows tended to be higher compared with that in vehicle-treated oocytes from young cows (pair wise comparison using the Students’ *t*-test, *P* = 0.038). In accordance with this, it has been suggested that mitochondrial quality and quantity is lower in aged cows than in young cows [15]. Moreover, ROS levels were found to be high in the oocytes of aged cows that underwent *in vitro* maturation and genes associated with mitochondria were differentially expressed in these oocytes between young and aged cows [17]. In subsequent experiments, we addressed whether oocyte mitochondrial biogenesis and degeneration in response to CCCP treatment differed between young and aged cows. To address mitochondrial biogenesis in oocytes, we used the proteasome inhibitor MG132, which inhibits the initial step of mitochondrial degeneration [31], allowing only mitochondrial biogenesis to occur (Fig 1). As we predicted, culturing COCs with MG132 for 21 h increased the levels of ubiquitinated proteins in oocytes of both aged groups. However, MG132 significantly increased MtDNA copy number in oocytes of young cows. MG132 has previously been reported to induce an increase in MtDNA copy number in in gilt oocytes [23, 24]. From these results, we suggested that mitochondrial degeneration and biogenesis are active in oocytes of young cows, but this is not the case in aged cows. When oocytes were treated with CCCP, mitochondrial biogenesis and degradation were upregulated in oocytes derived from young cows, such that the MtDNA copy number increased significantly following incubation in the medium that only supported mitochondrial biogenesis, whereas the MtDNA copy number did not differ in the medium that supported both mitochondrial generation and degeneration. Interestingly, this trend was not observed in oocytes derived from aged cows (summarized in Fig 9). This result led us to hypothesize that in response to CCCP-induced mitochondrial dysfunction, mitochondrial biogenesis and degeneration is upregulated in oocytes of young, but not aged cows.

SIRT1-PGC1a and TFAM pathways regulate mitochondrial biogenesis [32]; and upregulation of SIRT1 expression by Res treatment enhanced mitochondrial biogenesis and degradation in porcine oocytes [24]. Furthermore, CCCP treatment of porcine oocytes also increased mitochondrial biogenesis and enhanced the level of SIRT1 and *TFAM* expression [23], while expression of *PGC1a* was unaffected. In this context, we addressed whether oocyte SIRT1 expression differed between young and aged cows following CCCP treatment. Immediately following CCCP treatment, SIRT1 expression was comparable between CCCP-treated and untreated oocytes, regardless of the age of the donor. However, 3 h after CCCP treatment, the SIRT1 expression in young cows was greater than that in vehicle-treated oocytes, whereas no upregulation was observed in the oocytes of aged cows. It could not be ruled out that the oocytes of aged cows might respond more slowly to CCCP treatment than those of younger cows; therefore, we compared SIRT1 expression 6 h after CCCP treatment but observed no differences between the CCCP-treated and untreated oocytes (S3 Fig). In mouse oocytes, a differential SIRT1 response to oxidative stress has been reported. Here, *SIRT1* mRNA levels were higher in the oocytes of aged mice compared with that in their younger counterparts and, in response to oxidative stress induced by H_2_O_2_ treatment, *SIRT1* expression was upregulated only in the oocytes of younger mice [35]. Here, we demonstrated that the basal SIRT1 protein level in oocytes was higher for aged cows, and this trend was also observed in our previous study [17]. That study, however, demonstrated that the mRNA expression of *SIRT1* and *PGC1a* in aged cows was 1.01 and 0.75-fold, respectively, that in young cows. This indicates that the protein levels of SIRT1 were differentially regulated compared to the mRNA levels. Together with those results, the present study revealed low level of mitochondrial biogenesis as well as low activation of mitochondrial biogenesis in response to induced mitochondrial dysfunction in oocytes of aged cows. We hypothesized that high ROS and SIRT1 levels in oocytes of aged cows hamper proper activation of mitochondrial quality control system in oocytes and that might cause aged-associated decline in oocyte quality.

In conclusion, CCCP-induced mitochondrial dysfunction activates mitochondrial biogenesis in the oocytes of young cows but this resilience is lost in the oocytes of aged cows.

## Supporting information

S1 Fig Effectiveness of the immunostaining of SIRT1. Oocytes were cultured with the primary antibody (SIRT1: 2 μg/mL IgG, Santa Cruz Biotechnology, Santa Cruz, CA) or the primary antibody and a SIRT1-peptide (Abcam 7770–100, 2 μg/mL or 10 μg/mL). Fluorescent intensity significantly decreased in a peptide-concentration-dependent manner.
(PDF)

S2 Fig Effect of CCCP on ATP content of denuded oocytes. CCCP treatment of denuded oocytes for 2h reduced ATP content in oocytes (young: pre-CCCP treatment 1.44 ± 0.2, after CCCP treatment 0.13 ± 0.02; aged: pre-CCCP treatment 1.34 ± 0.22, after CCCP treatment 0.30 ± 0.11, P < 0.01, S2 Fig).
(PDF)

S3 Fig SIRT1 expression levels in oocytes of aged cows 6h after the CCCP treatment. Approximately 20 COCs were randomly selected from pooled COCs harvested from aged cows, and divided into two groups that were treated with either the vehicle or 10 μM of CCCP for 2 h, and then, the SIRT1 levels were examined 6 h after treatment. This experiment was repeated two times.
(PDF)
