# Supplementary figures and images for: Maternal aging affects oocyte resilience to carbonyl cyanide-m-chlorophenylhydrazone -induced mitochondrial dysfunction in cows
Source: PLoS One. 2017 Nov 28;12(11):e0188099. doi: 10.1371/journal.pone.0188099 (PMC5705080; doi:10.1371/journal.pone.0188099)

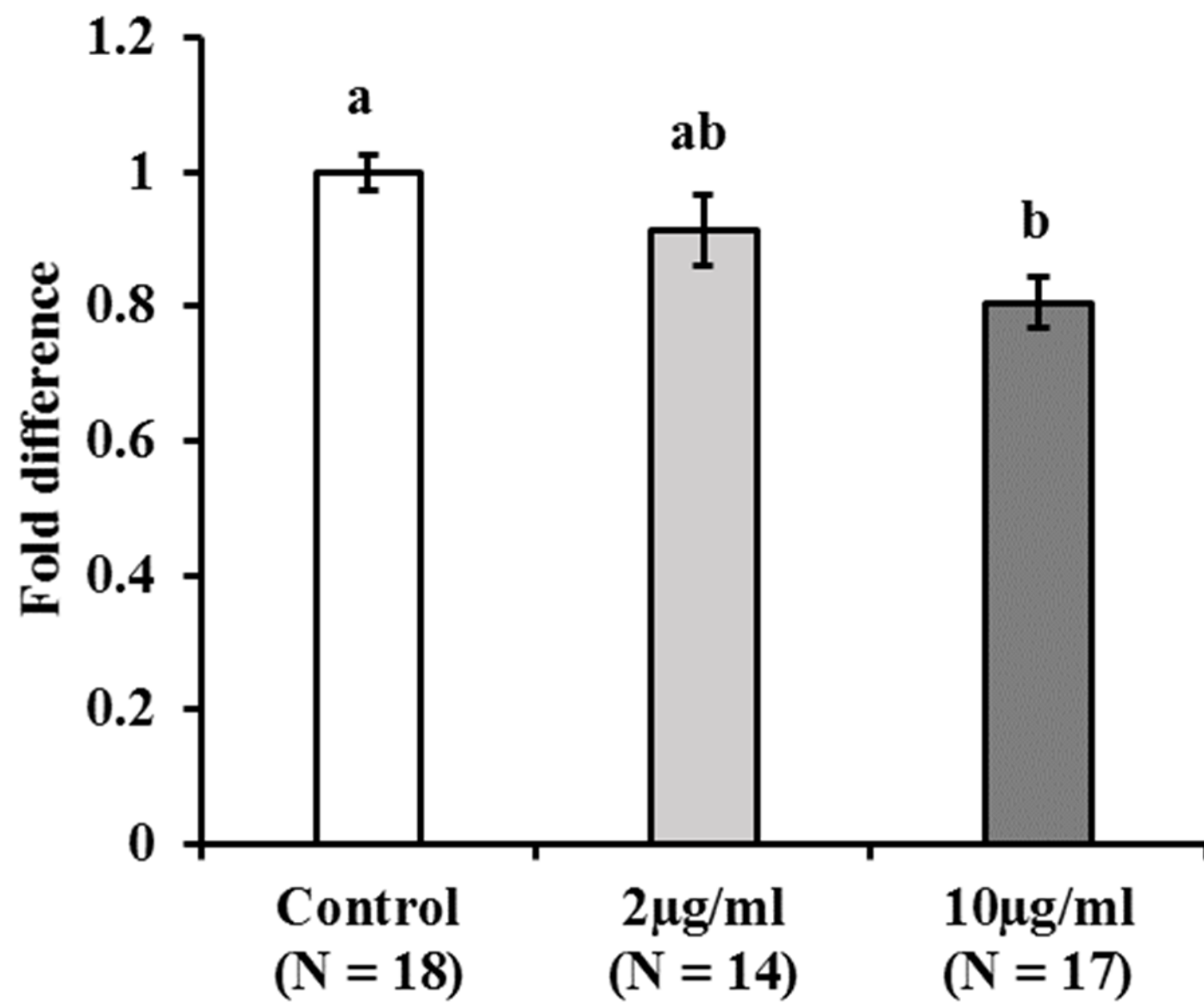

Supplement: S1 Fig — Oocytes were cultured with the primary antibody (SIRT1: 2 μg/mL IgG, Santa Cruz Biotechnology, Santa Cruz, CA) or the primary antibody and a SIRT1-peptide (Abcam 7770–100, 2 μg/mL or 10 μg/mL). Fluorescent intensity significantly decreased in a peptide-concentration-dependent manner. (PDF) [file pone.0188099.s001.pdf]

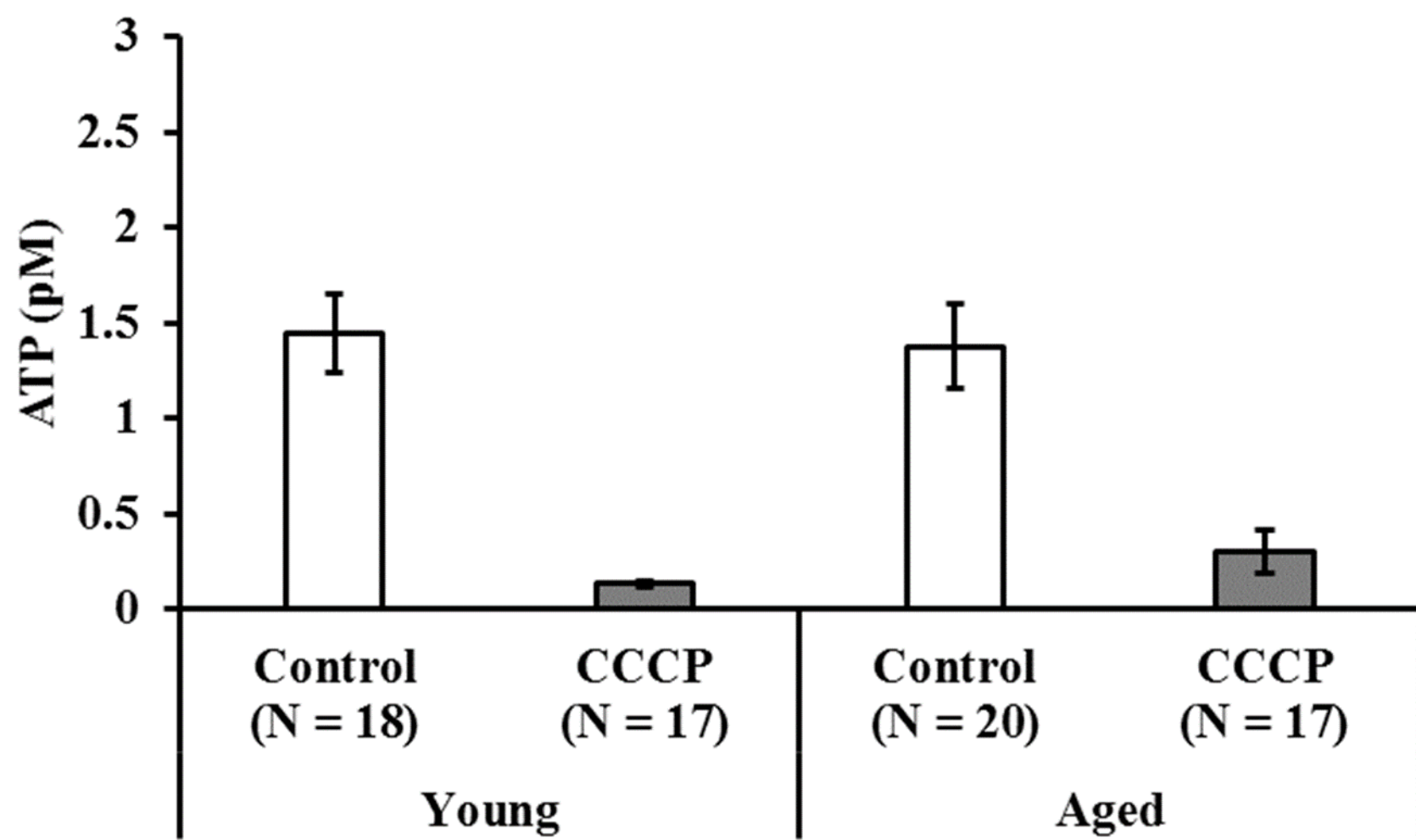

Supplement: S2 Fig — CCCP treatment of denuded oocytes for 2h reduced ATP content in oocytes (young: pre-CCCP treatment 1.44 ± 0.2, after CCCP treatment 0.13 ± 0.02; aged: pre-CCCP treatment 1.34 ± 0.22, after CCCP treatment 0.30 ± 0.11, P < 0.01, S2 Fig). (PDF) [file pone.0188099.s002.pdf]

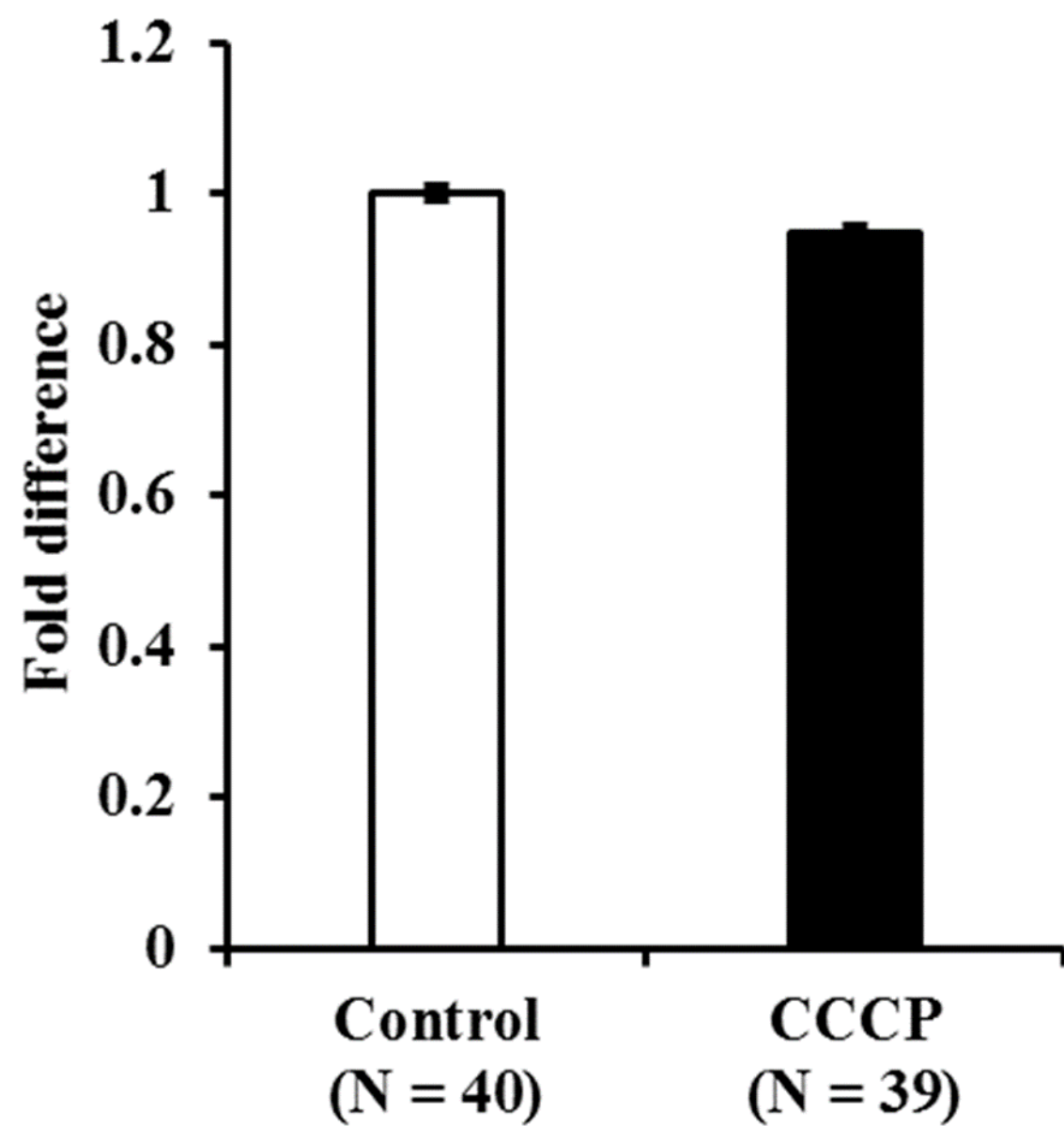

Supplement: S3 Fig — Approximately 20 COCs were randomly selected from pooled COCs harvested from aged cows, and divided into two groups that were treated with either the vehicle or 10 μM of CCCP for 2 h, and then, the SIRT1 levels were examined 6 h after treatment. This experiment was repeated two times. (PDF) [file pone.0188099.s003.pdf]
